# Effect of Bergamot and Laoxianghuang Polysaccharides on Gut Microbiota Derived from Patients with Hyperlipidemia: An Integrative Analysis of Microbiome and Metabolome during In Vitro Fermentation

**DOI:** 10.3390/foods11142039

**Published:** 2022-07-10

**Authors:** Yang Zheng, Yi Wang, Donghui Luo, Lianzhu Lin, Xingyu Lu, Jie Gao, Chuqiao Xiao, Mouming Zhao

**Affiliations:** 1 Chaozhou Branch of Chemistry and Chemical Engineering Guangdong Laboratory, Chaozhou 521000, China; an2zhengyang@126.com (Y.Z.); luodonghui@gdou.edu.cn (D.L.); felzlin@scut.edu.cn (L.L.); 2 School of Food Science and Engineering, South China University of Technology, Guangzhou 510640, China; 3 Jinhua Academy of Agricultural Sciences, Jinhua 321000, China; nazard@163.com; 4 College of Food Science and Engineering, Guangdong Ocean University, Yangjiang 529500, China; 5 School of Light Industry and Food Engineering, Guangxi University, Nanning 530004, China; 2016301019@st.gxu.edu.cn (X.L.); gaojie@gxu.edu.cn (J.G.)

**Keywords:** polysaccharide, hyperlipidemic, gut microbiota, SCFAs, metabolomics

## Abstract

The aim of this study was to investigate the effects of bergamot polysaccharide (BP) and Laoxianghuang polysaccharides (LPs, fermented bergamot) on the microbiome and metabolome during the in vitro fermentation of gut microbiota from patients with hyperlipidemia. Results indicated that both BP and LPs were able to increase the production of acetic acid, propionic acid, and butyric acid. However, only LPs could decrease the content of isobutyric acid and isovaleric acid, which are detrimental to gut health. A 16S rRNA analysis showed that both BP and LPs could reduce the proportion of *Fusobacterium*, whereas they increased the *Bacteroides* content in hyperlipidemia. Untargeted UPLC-MS/MS metabolomic profiling found six bio-markers that were significantly changed after BP and LPs intervention, and four of the down-regulated metabolites were long-chain fatty acids associated with vascular diseases. These findings provide new evidence that BP and LPs have the potential to regulate imbalances in the gut microbiota in patients with hyperlipidemia and ameliorate its metabolic abnormalities.

## 1. Introduction

Hyperlipidemia is a common metabolic syndrome that is characterized by abnormal blood lipids, manifested by elevated levels of total cholesterol, triglyceride, and low-density lipoprotein cholesterol and a decreasing level of high-density lipoprotein cholesterol [1]. It can induce atherosclerosis, which is a major risk factor for cardiovascular and cerebrovascular diseases, such as coronary heart disease, stroke, and myocardial infarction [2]. Hyperlipidemia is also related to the occurrence and development of obesity-related chronic diseases, such as diabetes, hypertension, and fatty liver [3]. Studies have also demonstrated the critical role of the gut microbiota in the development of obesity-related diseases [4]. Although lipid-lowering chemical drugs such as niacins, fibrates, and statins have been widely used in the regulation of the gut microbiota and the treatment of hyperlipidemia, these drugs also have limitations and may have side-effects, including myalgia and decreasing renal function [5,6]. As a result, multifunctional natural plants have received extensive interest as alternative therapies to cure hyperlipidemia due to their safe properties. Specifically, the anti-hyperlipidemia activity of Chinese herbal medicines and medicine food homologous plants have attracted widespread attention [7,8].

Bergamot (*Citrus medica* L. var. *sarcodactylis Swingle*), belonging to the Rutaceae family, has a long history of cultivation and medicinal use in China [9]. It contains various bioactive substances (bergamot oil, polysaccharides, hesperidin, alkaloids, and coumarins, etc.) and can exert various biological activities, such as anti-oxidation and anti-tumor properties, regulating blood lipids, lowering blood pressure, and immune regulation [10,11,12,13]. For example, a previous study indicated that a new bergamot polysaccharide exhibited free-radical-scavenging properties and immunoregulatory activity [9]. Another study showed that bergamot oligosaccharide exhibited the potential to be used as an effective source of prebiotics, increasing the populations of probiotics [11]. Laoxianghuang is a kind of traditional fermented bergamot in China which is produced through complex steps, including cooking, salting, washing, drying, and sealing and fermentation [14]. It is generally applied in traditional Chinese medicine due to its beneficial effects in regulating gut function, promoting digestion, and relieving coughs [15]. Although bergamot and Laoxianghuang have been studied for several years, few studies have reported the potential of polysaccharides derived from bergamot and/or Laoxianghuang in the regulation of gut microbiota or for hyperlipidemia treatment.

Polysaccharides are macromolecules consisting of more than 10 monosaccharides units through glycosidic bonds, which are widely present in algae, plants, microorganisms, and animals [10]. In recent years, more and more studies on the separation and identification of bioactive polysaccharides from natural sources have been reported [16], indicating that polysaccharides can exhibit multiple biological functions, including anti-hyperlipidemic activity [17]. For instance, *Pleurotus ostreatus* polysaccharides ameliorate dyslipidemia through the regulation of lipid metabolism, including the metabolism of glycerophospholipids, fatty acids, prenol lipids, and sphingolipids [18]. *Holothuria leucospilota* polysaccharides elevate the level of short-chain fatty acids to regulate lipid metabolism and subsequently relieve hyperlipidemia [19]. Auricularia auricula polysaccharides have been reported to improve the intestinal microbial environment by enriching SCFA (short-chain fatty acids)-producing bacteria, leading to the up-regulation of SCFAs and subsequently resulting in the relief of liver damage and hyperlipidemia in rats [20]. However, to date, few studies on the structure and biological properties of bergamot and Laoxianghuang-derived polysaccharides have been reported.

Consequently, in this study, BP and LPs were used as an intervention for the in vitro fermentation of feces derived from the general population and patients with hyperlipidemia. Changes in bacterial community structure, SCFAs, and non-targeted metabolites during fermentation were assessed using 16S rRNA, GC-MS, and UPLC-MS/MS, respectively, aiming to explore the effect of BP and LPs on regulating the gut microbiome and metabolome in hyperlipidemia.

## 2. Materials and Methods

### 2.1. Reagents and Materials

Fresh bergamot fruits and Laoxianghuang were purchased from the local market in Chaozhou, China. Standards of monosaccharide were purchased from Shanghai Yuanye Bio-Technology Co., Ltd. (Shanghai, China). Standards of dextran were purchased from Sigma-Aldrich Chemical Co., Ltd. (St. Louis, MO, USA). Standards of SCFAs (formic acid, acetic acid, propionic acid, butyric acid, valeric acid, isobutyric acid, and isovaleric acid) were purchased from Shanghai Aladdin Biochemical Technology Co., Ltd. (Shanghai, China). Ethyl acetate of HPLC grade was supplied by Chengdu Kelong chemical Co., Ltd. (Sichuan, China), and methanol of HPLC grade was supplied by Tianjin shield special chemical Co., Ltd. (Tianjin, China). Other reagents were of analytical grade.

### 2.2. Extraction of Polysaccharides

Fresh bergamot slices were dried at 40 °C then crushed into a powder. The bergamot powder was added into pure water at a ratio of 1:10 (*w*/*v*) and extracted at 80 °C for 5 h [21]. The supernatant was concentrated by vacuum rotary evaporation at 60 °C and then mixed with 95% ethanol to an ethanol concentration of 80%. The mixture was placed at 4 °C for 24 h before centrifugation at 8000× *g* rpm for 15 min. In order to remove protein, the sediment was mixed with 4% trichloroacetic acid (*w*/*v*) and placed at 4 °C for 10 h. The supernatant was collected and dialyzed in dialysis bags for 2 days at 4 °C to remove the residual trichloroacetic acid. Then, the solution was lyophilized to obtain BP.

LPs was extracted in a similar way. In brief, 10 g of Laoxianghuang was added to 100 mL pure water and then homogenized before being extracted at 80 °C for 5 h. The following steps were performed according to the BP, as mentioned previously. Laoxianghuang fermented for 1, 3, and 5 years was named mono-Laoxianghuang, tri-Laoxianghuang, and penta-Laoxianghuang. The polysaccharides derived from mono-Laoxianghuang, tri-Laoxianghuang, and penta-Laoxianghuang were denoted as MLP, TLP, and PLP, respectively.

### 2.3. Monosaccharide Composition

The polysaccharides were hydrolyzed with trifluoroacetic acid and then derivatized by 1-phenyl-3-methyl-5-pyrazolone (PMP) [22]. The free PMP was removed by chloroform. The composition of monosaccharides in polysaccharides was determined by a HPLC instrument equipped with a Xbridge C18 column (15 cm × 0.2 mm × 0.25 μm; Waters Co., Milford, MA, USA). PBS buffer (0.02 mol/L, pH = 6.7) and acetonitrile mixture solution (83:17, *v*/*v*) were used as the mobile phase. Samples (10 mg/mL, 20 uL) were injected and eluted at 30 °C under a flow rate of 1 mL/min for 60 min. The detector collects the response value at a wavelength of 245 nm. Quantification was achieved by plotting a calibration curve with standards for D-Mannuronic acid (Man-A), D-Mannose (Man), L-Rhamnose (Rha), D-Glucuronic acid (Glc-A), D-Galacturonic acid (Gal-A), D-Glucose (Glc), D-Galactose (Gal), D-Arabinose (Ara), and L-Fucose (Fuc).

### 2.4. Determination of Molecular Weight

An Agilent-1260 high-performance liquid chromatograph system (Agilent Tech., California, CA, USA) equipped with TSK-G2500PWXL and TSK-G5000PWXL (15 cm × 0.2 mm × 0.25 μm; TOSOH Bioscience, Tokyo, Japan) columns was utilized to determine the molecular weight of polysaccharides [23]. Ultra-pure water was used as a mobile phase at a constant flow rate of 0.5 mL/min. The injection volume was 20 μL and the column incubator was maintained at 30 °C. Dextran of different molecular weights (1, 5, 12, 25, 50, 80, 150, 270, 410, and 670 kDa) was applied as standards.

### 2.5. In Vitro Fermentation

This study was approved by the Guangxi University Ethics Committee (approval no.: GXU-2021144), and the volunteers signed informed consent forms. The fecal samples were taken from 4 volunteers with hyperlipidemia and 4 healthy people (two males and two females aged from 20 to 40). About 5 g of each fecal sample was quickly placed in a centrifuge tube containing liquid paraffin and normal saline. The tubes were then packed in sealed bags and preserved at −80 °C before being used within three days.

The fundamental anaerobic culture medium (FAM) was prepared as described before, with slight modifications [24]. The FAM was composed of 1.6 mmol K_2_HPO_4_, 7.5 mmol NaCl, 1.3 mmol KH_2_PO_4_, 0.4 mmol CaCl_2_, 3.4 mmol (NH_4_)_2_SO_4_, 0.7 mmol MgSO_4_, 4.0 mmol L-cysteine, 2.8 mmol L-ascorbic acids, 37.7 mmol Na_2_CO_3_, 1 g nutrient agar, 1 g beef extract, and 1 g peptone in 1 L water. The pH of the FAM was controlled at 7.5–8.0.

Before fermentation, the fecal samples were fully mixed, followed by centrifugation at 500× *g* rpm for 3 min. Under anaerobic conditions, the bacterial liquid under the liquid paraffin was inserted into different anaerobic culture mediums with 10% inoculum. According to the differences in feces and mediums, we created six groups: The feces of the healthy population were inoculated in FAM as a normal control group (NC); the feces of hyperlipidemic people were inoculated in FAM as a hyperlipidemia control group (HC); the feces of hyperlipidemic people were inoculated in FAMs with 0.5% of different polysaccharides (BP, MLP, TLP, and PLP). In each group, a total of 3 parallel tubes was used. All tubes were placed on the tube rack and cultured anaerobically under continuous shaking at 37 °C for 48 h. Samples were taken every 12 h and repeated three times at each point (0 h, 12 h, 24 h, 36 h, and 48 h) for the detection of short-chain fatty acids, microbial structure, and metabolites.

### 2.6. Short-Chain Fatty Acids (SCFAs) Analysis

For each tube, 500 μL of sample was mixed with 5 μL of formic acid standard before being stored at −20 °C for more than 2 h. After that, the mixture was centrifuged at 15,000× *g* rpm for 2 min after thawing. Then, 400 μL of supernatant was taken and extracted using 400 μL of ethyl acetate for 2 min, followed by centrifugation at 8000× *g* rpm for 10 min. A total of 300 μL of supernatant was taken and mingled with 3 μL of 4-methyl valeric acid (5.85 mg/mL). The ethyl acetate phase was obtained and filtered by a nylon filter (0.22 μm).

The pretreated samples were analyzed using the GC-MS system equipped with GC (Agilent 7890B mag Agilent Technologies, Santa Clara, CA, USA) and highly inert MSD (Agilent 5977A mag Agilent Technologies, Santa Clara, CA, USA) [25]. The separation was carried out on a TG-WAXMS column (60 m × 0.25 mm × 0.25 μm, Thermo Science, Waltham. MA, USA). The injector was kept at 250 °C and the flow rate of helium was 1 mL/min. The temperature program started at 90 °C and rose to 150 °C at the rate of 15 °C/min, then to 170 °C at the rate of 5 °C/min, and finally to 250 °C at the rate of 20 °C/min, before holding for 2 min. The injection volume was 1 μL and all samples were analyzed with a split ratio of 1:1.

### 2.7. Metabolomics Analysis

A total of 500 μL of sample was mixed with 3000 µL of methanol (HPLC grade) and vortexed for 1 min. The mixture was centrifuged at 13,000× *g* rpm for 10 min and filtered using a 0.22 µm filter before analysis [26,27]. UPLC-MS/MS analysis was conducted using a Q Exactive Plus system (Thermo-fisher Scientific, MA, USA) equipped with a Hypersil GOLD C18 column (100 × 2.1 mm, 1.9 μm, Thermo-fisher Scientific). 

The mobile phase was composed of 0.1% formic acid aqueous solution (A) and acetonitrile (B). The fractions were eluted at a flow rate of 0.30 mL/min using the following conditions: 5% of solvent B for 2 min, which was linearly increased from 5 to 95% within 14 min, then 5% of solvent B for 2 min. MS analysis was conducted using an electrospray ionization (ESI) source at 3.0 kV operated in both positive and negative mode. The mass spectra were collected from 100 to 1000 *m*/*z* under a resolution of 70,000 FWHM. The target of Automatic Gain Control (AGC) was 3 × 10^6^ within 100 ms. For dd-MS2, the mass spectra were recorded at a resolution of 17500 FWHM, with a AGC value of 1 × 10^5^ within 50 ms. Fragment ions were generated in HCD collision cells using stepped normalized collision energy (NCE 10, 25, and 45%). The results obtained were analyzed using the Compound Discoverer software (Thermo Scientific, MA, USA).

### 2.8. 16S rRNA Amplicon Sequencing of Microbiota

Microbial DNA was extracted using the HiPure Soil DNA Kit (Magen, Guangzhou, China) according to the manufacturer’s protocols. The 16S rDNA target region of the ribosomal RNA gene was amplified by polymerase chain reaction (PCR) using 341F (CCTACGGGNGGCWGCAG) and 806R (GGACTACHVGGGTATCTAAT) as primers [28]. PCR reactions were performed in triplicate.

Raw data containing adapter or low-quality readings will affect subsequent assembly and analysis. Hence, FASTP (version 0.18.0, HaploX, Shenzhen, China) [29] and FLASH (version 1.2.11, the Center for Computational Biology at Johns Hopkins University, MD, USA) [30] were used to combine the paired-end clean readings with the original label, with a minimum overlap of 10 BP and a mismatch error rate of 2%. The noise sequence of the original tag was filtered under specific filtering conditions to obtain high-quality clean tags [31]. The UPARSE (version 9.2.64) pipeline was applied to cluster clean tags into operational taxonomic units (OTUs) with a similarity ≥ 97% [32]. All chimeric tags were removed by the UCHIME algorithm, and effective tags were obtained for further analysis. In each cluster, the tag sequence with the highest abundance was selected as the representative sequence.

### 2.9. Statistical Analysis

The data were expressed as mean ± standard deviation (n = 3) of repeated analyses. Analysis of variance (ANOVA) was performed and statistical analyses was performed using software including R statistical package, XCMS Online, Metaboanalyst 5.0, and Omicsmart.

## 3. Results and Discussion

### 3.1. Structural Characterization

The biological activities of polysaccharides are closely related to their structural characteristics, such as monosaccharide composition and molecular weight [33]. The monosaccharide compositions of BP and LPs are listed in Table 1. As can be seen, BP was composed mainly of Man, Rha, and Gal-A. However, significant differences were observed in LPs, which consisted of Gal-A, Gal, Rha, Fuc, and Glc-A. Compared with BP, LPs had more complex monosaccharide compositions. This is similar to the finding of a previous study in that with the extension of the storage period of Chenpi, the number of types of monosaccharides increased in the polysaccharide [34]. The content of Man and Rha in BP was much higher than that in the LPs, whereas the monosaccharide compositions present in the LPs were typical for pectic polysaccharides, which mainly consisted of Rha, Gal, and Gal-A [35]. Moreover, the contents of monosaccharides of LP groups (MLP, TLP, and PLP) were different from each other, which may be associated with the long-term action of various external materials and microorganisms during fermentation.

HPLC analysis revealed that the molecular weight (MW) of BP (2.0 × 10^7^ Da) was higher than that of MLP (6.9 × 10^6^ Da), TLP (4.7 × 10^6^ Da), and PLP (5.6 × 10^6^ Da). However, minor differences were found among the LPs, indicating that fermentation time may not be an important factor affecting the molecular weight distribution of LPs (Figure S1). 

### 3.2. SCFAs Production in the Vitro Fermentation

**Figure 1:**
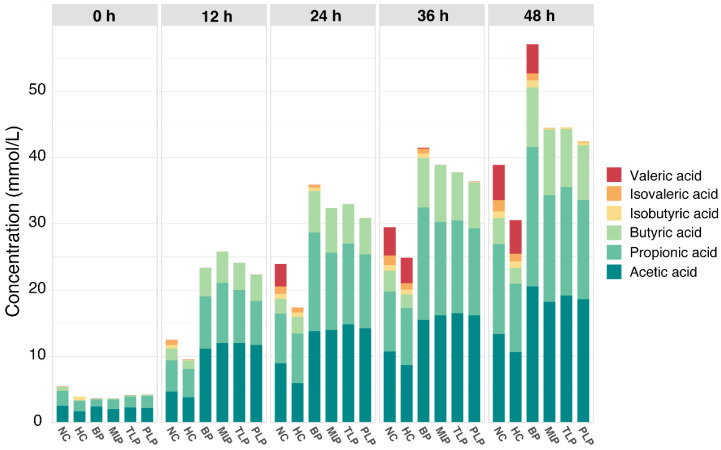
The levels of SCFAs in different groups and time during in vitro culture. NC: the feces of the healthy population were inoculated in FAM; HC: the feces of the hyperlipidemic people were inoculated in FAM; BP, MLP, TLP, PLP: the feces of the hyperlipidemic people were inoculated in FAMs with 0.5% of BP, MLP, TLP, and PLP. All experiments were repeated in triplicate (n = 3).

SCFAs are important metabolites that play a vital role in human health [36]. Figure 1 shows the content of SCFAs during the in vitro fermentation of the gut microbiota as affected by different polysaccharides. As can be seen, the SCFAs were mainly composed of acetic acid, propionic acid, butyric acid, isobutyric acid, and isovaleric acid, among which acetic acid, propionic acid, and butyric acid were the most abundant, whereas isovaleric acid was the least. Overall, the content of SCFAs increased along with the fermentation time in a time-dependent manner. Compared with the NC group, the HC group exhibited a significantly lower production of SCFAs; however, the groups treated with polysaccharides showed higher levels of production. After 48 h of fermentation, the BP group produced the most SCFAs, followed by the MLP, TLP, and PLP groups (Figure 1). These results suggested that hyperlipidemia may inhibit the production of SCFAs in the gut, while the intervention with BP and LPs tended to stimulate SCFA production, especially for BP. Many studies have indicated that SCFAs, especially acetic acid, propionic acid, and butyric acid, can exert multiple bioactive functions and protect host health through promoting weight loss, glycemic control, and anti-hyperlipidemia [37,38]. However, branch chain fatty acids, including isobutyric acid and isovaleric acid, have been shown to alter the cellular morphology of the gut and be detrimental to gut health [39,40]. In the present study, acetic acid, propionic acid, and butyric acid were the major SCFAs in all groups. Furthermore, the levels of isobutyric acid and isovaleric acid were very low, especially in the LP groups. In this regard, BP and LPs can reverse the change in SCFAs caused by hyperlipidemia. This result is in accordance with that of previous studies, in which Holothuria Leucospilota polysaccharide was found to improve the levels of SCFAs to affect lipid metabolism and then alleviate hyperlipidemia [19]. In addition, the content of acetic acid and propionic acid had the same trend as that for the total SCFAs in the different groups (Figure S2), indicating the promising response of these fatty acids to fermentation. However, minor changes in the content of SCFAs in the MLP, TLP, and PLP groups were observed, indicating that fermentation time had little effect on the production of SCFAs during fermentation. The results obtained suggested the potential of BP and LPs to up-regulate the production of SCFAs in patients with hyperlipidemia, which is beneficial for the health of the organism.

### 3.3. Changes of Gut Microbiota

**Figure 2:**
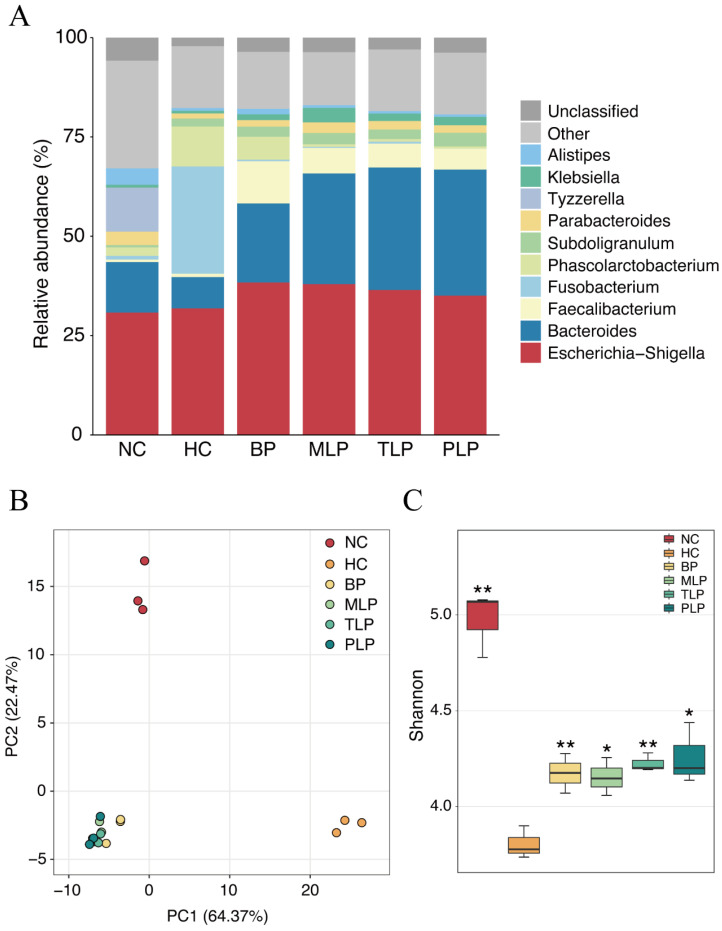
Microbial changes in different groups after 48 h of fermentation in vitro. (**A**) The relative abundance of the main taxa at the genus level. The beta diversity obtained using the (**B**) PCA analysis. (**C**) Shannon index demonstrated the alpha diversity. Differences from the HC group were assessed using Student’s *t* test and are denoted as follows: * *p* < 0.05, ** *p* < 0.01. NC: the feces of the healthy population were inoculated in FAM; HC: the feces of the hyperlipidemic people were inoculated in FAM; BP, MLP, TLP, PLP: the feces of the hyperlipidemic people were inoculated in FAMs with 0.5% of BP, MLP, TLP, and PLP. All experiments were repeated in triplicate (n = 3).

Bacteria is a main factor that influences fermentation and related metabolites [41]. In this study, the gut microbiota was analyzed after 48 h of fermentation according to our previous study [24]. To identify the species of bacteria in the different groups, the relative abundance of bacteria in different groups was compared at the genus level. As shown in Figure 2A, Escherichia-Shigella was dominant in all groups, followed by Bacteroides, except for the HC group. Significant changes in the HC group were observed as compared with the NC group, mainly including a decreased abundance of Bacteroides and an increased content of Fusobaterrium and Phascolarctobaterium species. With the treatments of BP and LPs, the abundance of Bacteroides and Faecalibacterium increased compared with that of the HC group, accompanied by the decrease in Fusobacterium and Phascolarctobacterium species (Figure 2A), which was inconsistent with the literature [20,42]. Moreover, Bacteroides has been reported to contain bile salt hydrolases that metabolize conjugated bile acids to unconjugated bile acids and thus act through the bile acid signaling pathway [43]. Additionally, Fusobacterium promotes inflammatory responses and colorectal carcinogenesis via its FadA adhesin [44]. Nevertheless, the MLP, TLP, and PLP groups exhibited similar structures of their bacterial community without any marked difference. These results indicated that the gut microbiota of people with hyperlipidemia will have significant differences compared with those of healthy individuals, and that treatment with BP and LPs may partially rectify the changes in the gut microbiota associated with hyperlipidemia.

A principal component analysis (PCA) was conducted to compare the operational taxonomic units (OTU) structure and community diversity of the gut microbiota in the five groups (Figure 2B). The results exhibited three statistical clusters among the NC and HC groups and the other four groups treated with BP and LPs, suggesting that hyperlipidemia and further intervention by polysaccharides resulted in differences in the gut microbiota during fermentation. In the PCA model, the plot explained 64.37 and 22.4% of variables by PC1 and PC2, respectively, indicating that the profiles were credible. Furthermore, the BP, MLP, TLP, and PLP groups showed a trend that was close to that of the NC group at the PC1 level. These results illustrated a visible difference in gut flora between the healthy population and the patients with hyperlipidemia, showing that the intervention with BP and LPs could reverse the changes in the gut microbiota of the hyperlipidemia group to bring them in line with the healthy population. 

Diversity of the gut microbiota is another key factor that is related to gut health and lipid metabolism. The Shannon index is an important parameter used for estimating species diversity [45]. In this study, the Shannon index was used to estimate the bacteria diversity difference among the six groups. As shown in Figure 2C, the Shannon index of the NC, BP, MLP, TLP, and PLP groups was significantly higher compared with that of the HC group, indicating the suppression of bacteria diversity by hyperlipidemia. Previous studies have demonstrated that more diverse bacteria flora tend to exist in the healthy population, whereas low bacterial diversity is more likely to be found in patients with dyslipidemia [45]. Hence, treatment with BP and LPs could reverse the diversity of gut bacteria, which is inhibited by hyperlipidemia, and exert beneficial effects on regulating dyslipidemia.

To identify the specific bacterial phylotypes that responded differently to the six groups, LEfSe analysis was applied with 4 used as a threshold value for the LDA score (Figure S3). The LDA score indicated that Bacteroidetes (Bacteroides) were the most differentially abundant bacteria in the TLP and PLP groups. Previous studies have shown that Bacteroidetes could use thousands of enzyme combinations to break down glycans and produce SCFAs [46,47]. In the BP and MLP groups, Faecalibacterium and Megamonas were the most abundant, respectively (LDA score > 4), and they could produce butyric acid and acetic acid, which are beneficial to health [48,49]. The results shown above illuminated the possible reason for the high content of SCFAs seen in the BP and LJP groups.

**Figure 3:**
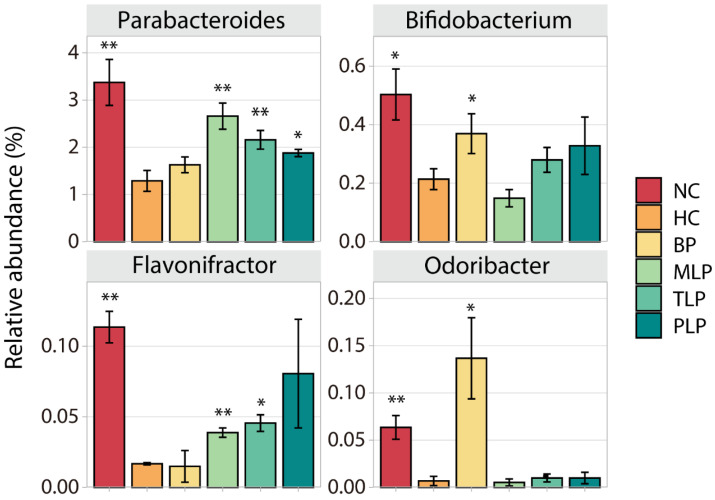
Changes in the content of four special bacteria in different groups after 48 h of fermentation in vitro. Differences in the HC group were assessed using Student’s *t* test and are denoted as follows: * *p* < 0.05, ** *p* < 0.01. NC: the feces of the healthy population were inoculated in FAM; HC: the feces of hyperlipidemic people were inoculated in FAM; BP, MLP, TLP, PLP: the feces of hyperlipidemic people were inoculated in FAMs with 0.5% of BP, MLP, TLP, and PLP. All experiments were repeated in triplicate (n = 3).

Different bacteria have different effects on the human body. To further investigate the effect of BP and LPs on the regulation of fecal microbiota in the hyperlipidemic population, we filtrated special bacteria according to the following principle: the contents of these were low/high in the HC group compared with the NC group, while they increased/decreased after the intervention with BP and/or LPs. In this regard, four widely studied species of bacteria (Parabacteroides, Bifidobacterium, Flavonifractor, and Odoribacter) were chosen for further analysis. As shown in Figure 3, significantly lower contents of all four species were observed in HC group as compared with the NC group, indicating the suppression of these bacteria by hyperlipidemia. However, intervention with BP or LPs could up-regulate their level to varying degrees. For example, BP significantly increased the contents of Bifidobacterium and Odoribacter, which produce short-chain fatty acids and regulate the immune response to maintain colonic health, prevent hypertension, and regulate blood glucose [50,51]. MLP and TLP could markedly enhance the level of Parabacteroides, which plays a positive role in dozens of diseases, such as inflammation and obesity [52]. The treatment with PLP resulted in a very high level of Flavonifractor, which is a butyric acid-producing bacteria that plays a beneficial role in health to some extent [53]. The above results demonstrate that the growth of certain bacteria which have potential health benefits was inhibited in the hyperlipidemia group, while the intervention with BP or LPs tended to reverse this inhibition. Moreover, BPs and LPs had different target bacteria, which is probably due to the diverse structures of BPs and LPs. The bacteria used different degrees of polysaccharides, which caused different levels of growth of bacteria.

### 3.4. Analysis of Metabonomics

To understand the beneficial effects of polysaccharides from the perspective of metabolism, untargeted metabolomics was used to analyze the fecal metabolome. PCA profiles provided an overall view of the metabolic differences among the different groups (Figure S4). The profiles of subjects in the MLP, TLP, and PLP groups were extremely similar, indicating that their metabolite profiles were similar. As for the NC, HC, and BP groups, the PCA plots showed obvious differences. It can be seen that the metabolites of individuals with hyperlipidemia are obviously different from those of the healthy population, and the intervention with BP and LPs altered the flora metabolism of the hyperlipidemia group.

**Figure 4:**
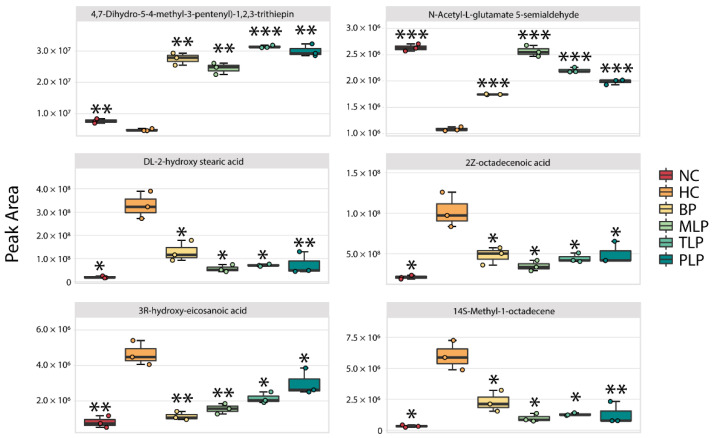
Changes in the content of six core metabolites in different groups after 48 h of fermentation in vitro. Differences from the HC group were assessed using Student’s *t* test and are denoted as follows: * *p* < 0.05, ** *p* < 0.01, *** *p* < 0.001. NC: the feces of the healthy population was inoculated in FAM; HC: the feces of hyperlipidemia people was inoculated in FAM; BP, MLP, TLP, PLP: the feces of hyperlipidemia people were inoculated in FAMs with 0.5% of BP, MLP, TLP, and PLP. All experiments were repeated triply (n = 3).

By filtering with a specific range of fold change values and p values (fold change of <0.5 or >2 and *p* value < 0.05), collections of metabolites with significant responses to the different mediums were obtained (Figure S5). Compared with the NC group, a total of 91 metabolites were significantly down-regulated and 79 metabolites were up-regulated in the HC group. The intervention with BP and LJPs regulated the production of metabolites to varying degrees. Among the significantly changed metabolites, six core metabolites were chosen for further analysis according to the same principle of the filtering of special bacteria (Figure 4). Compared with the NC group, significantly lower contents of 4,7-Dihydro-5-(4-methyl-3-pentenyl)-1,2,3-trithiepin and N-Acetyl-L-glutamate 5-semialdehyde and higher contents of DL-2-hydroxy stearic acid, 2Z-octadecenoic acid, 3R-hydroxy-eicosanoic acid, and 2-hydroxyhexadecanoic acid were observed in the HC group. Among these metabolites, N-Acetyl-L-glutamate 5-semialdehyde is a type of glutamate that can reduce oxidative stress and protect the intestinal function [54]. However, DL-2-hydroxy stearic acid, 2Z-octadecenoic acid, 3R-hydroxy-eicosanoic acid, and 2-hydroxyhexadecanoic acid are long-chain fatty acids (LCFAs) which have been reported to exert harmful effects, including accelerating the formation of fatty liver and obesity [55,56]. In this regard, hyperlipidemia tended to suppress the production of beneficial metabolites but, conversely, promoted the generation of harmful metabolites. Interestingly, the treatment with BP and LPs reversed the trends in metabolite production influenced by hyperlipidemia, regulating the metabolism of hyperlipidemic flora towards health.

**Figure 5:**
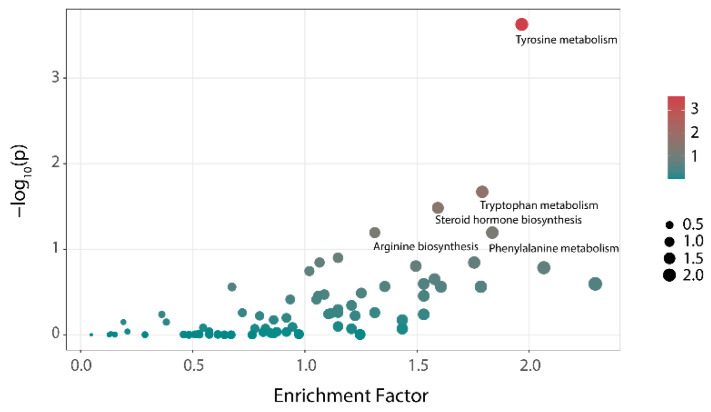
Analysis of the differences in metabolic pathways between the NC group and HC group (bubble color represents the hypergeometric test *p*-value of the metabolic pathway. Bubble size indicates the number of differential metabolites involved in this pathway).

The results of the differential metabolic pathways analysis of the NC and HC groups at 48 h are shown in Figure 5. Tyrosine metabolism, tryptophan metabolism, and steroid hormone biosynthesis showed significant differences among the two groups (*p* < 0.05). Additionally, these were the main metabolic pathways that distinguishes individuals with hyperlipidemia from healthy people and might be related to the formation of hyperlipemia. After treatment with polysaccharides, the three metabolic pathways showed almost no difference compared with that of the NC group (Table 2). That means that the differences in the metabolic pathways caused by hyperlipidemia could be reversed through the intervention with polysaccharides.

### 3.5. Correlation Analysis among SCFAs, Differential Metabolites, and Gut Microbiota

**Figure 6:**
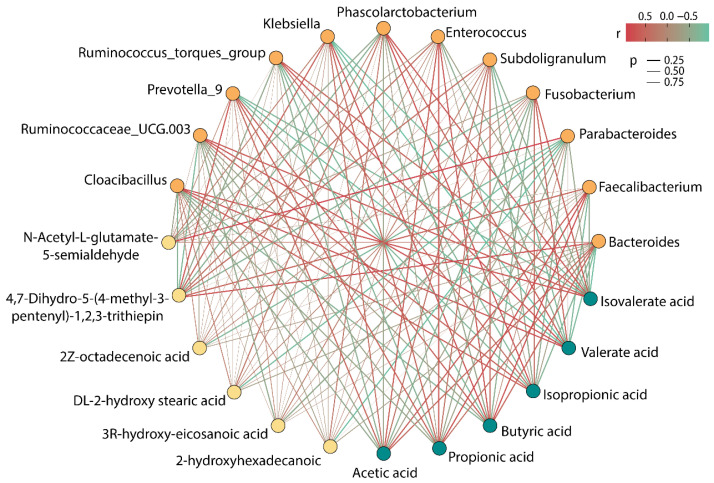
Correlation among gut microbiota, SCFAs, and differential metabolites (the color of dots signifies different categories, where the orange dot represents the microbiota, the green dot represents SCFAs, and the yellow dot represents metabolites).

Figure 6 showed the correlation among intestinal microbiota, SCFAs, and special metabolites. Parabacteroides, Fusobacterium, Phascolarctobacterium, Ruminococcus_torques_group, Ruminococcaceae_UCG.003, and Cloacibacillus showed a negative correlation with acetic acid, propionic acid, and butyric acid, while showing a positive correlation with isobutyric acid, isovalerate acid, and valerate acid. Bacteroides, Faecalibacterium, Subdoligranulum, Enterococcus, Klebsiella, and Prevotella_9 were found to have an obvious active effect on the production of acetic acid, propionic acid, and butyric acid, whereas they had an adverse effect on the production of isobutyric acid, isovalerate acid, and valerate acid. Thereof, Faecalibacterium had the strongest correlation with the production of acetic acid, propionic acid, and butyric acid, and its content was the highest in the six groups, which could explain the reason for the high rate of accumulation of SCFAs after 48 h of fermentation in the BP group. 

Moreover, it was worth noting that 4,7-Dihydro-5-(4-methyl-3-pentenyl)-1,2,3-trithiepin showed a strong positive correlation with Bacteroides and Prevotella_9 and showed a negative correlation with Ruminococcaceae_UCG.003 and Cloacibacillus. N-Acetyl-L-glutamate 5-semialdehyde exhibited a strong positive association with Parabacteroides. Astonishingly, *Parabacteroides* demonstrated a powerful negative correlation with four LCFAs, and it might be a bacterium that deserves our attention in subsequent research.

## 4. Conclusions

In summary, the gut microbiota and its metabolites were significantly different in the healthy individuals and those with hyperlipidemia after 48 h of fermentation in vitro, and the treatment with BPs and LPs for hyperlipidemia could change the gut microbiota and metabolites. The content of SCFAs was lower in the hyperlipidemia group compared with the healthy group and showed a significant improvement after BP and LP intervention. Specially, only LPs could decrease the content of isobutyric acid and isovaleric acid, which are detrimental to gut health.

The PCA analysis of the structure of the bacterial community indicated the presence of remarkable differences between healthy individuals and those with hyperlipidemia. BPs and LPs affected the bacterial community of the group with hyperlipidemia and brought it closer to that of the healthy group. The metabolomics analysis demonstrated that the metabolites filtered from the healthy individuals and those with hyperlipidemia were extremely different. The levels of 4,7-Dihydro-5-(4-methyl-3-pentenyl)-1,2,3-trithiepin and N-Acetyl-L-glutamate 5-semialdehyde were lower in the hyperlipidemia group than in the healthy group. Four kinds of LCFAs, DL-2-hydroxy stearic acid, 2Z-octadecenoic acid, 3R-hydroxy-eicosanoic acid, and 2-hydroxyhexadecanoic acid, were found in significantly high quantities in the hyperlipidemia group. The levels of six core metabolites were altered and tended to improve health during the intervention with BP and LPs. Correlation analysis revealed that *Faecalibacterium* might be the reason for the high levels of SCFAs seen in the BP and LPs groups, and *Parabacteroides* could downgrade the production of LCFAs. Thus, a comprehensive analysis of colony structure and metabolomics showed that BP and LPs may affect the production of SCFAs and metabolites by changing the structure of the gut microbiota, leading to an improvement in intestinal health for the hyperlipidemia group. However, the specific mechanism by which lipids are regulated by BP and LPs is still unclear and needs to be verified by subsequent animal experiments.

## Figures and Tables

**Table 1:** Monosaccharide composition of BP and LPs.

Monosaccharide Composition/%	Man-A	Man	Rha	Glc-A	Gal-A	Glc	Gal	Ara	Fuc
BP	-	63.62	24.16	-	10.62	-	1.60	-	-
MLP	0.93	1.53	5.39	4.91	47.16	-	28.59	3.18	8.31
TLP	0.05	3.64	10.04	2.66	42.49	5.71	30.57	1.04	3.00
PLP	0.66	2.00	4.96	5.47	56.34	1.41	14.40	1.35	13.41

**Table 2:** Significant differences analysis of three metabolic pathways between the NC group and the other five groups.

*p* Value	Tyrosine Metabolism	Tryptophan Metabolism	Steroid Hormone Biosynthesis
HC	0.0002	0.0213	0.0328
BP	0.0108	0.1390	0.5402
MLP	0.0179	0.0220	0.9998
TLP	0.0922	0.2837	0.9953
PLP	0.1719	0.2156	0.9852

## Data Availability

Data is contained within the article.

## Supplementary Materials

The following supporting information can be downloaded at https://www.mdpi.com/article/10.3390/foods11142039/s1, Figure S1: Peak time diagram of BP and LPs. Figure S2: The levels of acetic, propionic acid, and butyric acid in different mediums and at different times during in vitro fermentation. Figure S3: Characterization of microbiomes in six groups by LEfSe analysis and LDA. (A) Taxonomic representation of statistically and biologically consistent differences in six groups. (B) Histogram shows the LDA scores (log10) identified for the size of differentiation between different groups with a threshold value of 4. Figure S4: Analysis of PCA differences in metabolomics after fermentation until 48 h. Figure S5. Volcano plot enabling the visualization of differential metabolites between different groups. (A) HC vs. NC. (B) BP vs. HC. (C) MLP vs. HC. (D) TLP vs. HC. (E) PLP vs. HC.
