# Supplementary material for: Effect of Bergamot and Laoxianghuang Polysaccharides on Gut Microbiota Derived from Patients with Hyperlipidemia: An Integrative Analysis of Microbiome and Metabolome during In Vitro Fermentation
Source: Foods. 2022 Jul 10;11(14):2039. doi: 10.3390/foods11142039 (PMC9323038; doi:10.3390/foods11142039)
Supplement: Supplementary file 1 [file foods-11-02039-s001.zip › foods-1762354-supplementary.pdf]

Supplemental data

Figure S1. Peak time diagram of BP and LPs.

Figure S2. The levels of acetic, propionic acid and butyric acid in different medium and time during in vitro fermentation. Differences from the HC group were assessed using analysis of Student's t test and are denoted as follows: *$p < 0.05$, **$p < 0.01$.

Figure S3. Characterization of microbiomes in six groups by LEfSe analysis and LDA. (A) Taxonomic representation of statistically and biologically consistent differences in six groups. (B) Histogram shows the LDA scores (log10) identified size of differentiation between different groups with a threshold value of 4.

Figure S4. Analysis of PCA differences in metabolomics after fermentation to 48 hours.

Figure S5. Volcano plot enabling the visualization of differential metabolites between different groups. (A) HC vs NC. (B)BP vs HC. (C)MLP vs HC. (D)TLP vs HC. (E)PLP vs HC. Metabolites with a fold change of <0.5 and a p value of <0.05 (highlighted in green) were denoted as significantly downregulated metabolites, while metabolites with a fold change of >2 and a *p* value of <0.05 (highlighted in red) represented significantly upregulated ones.

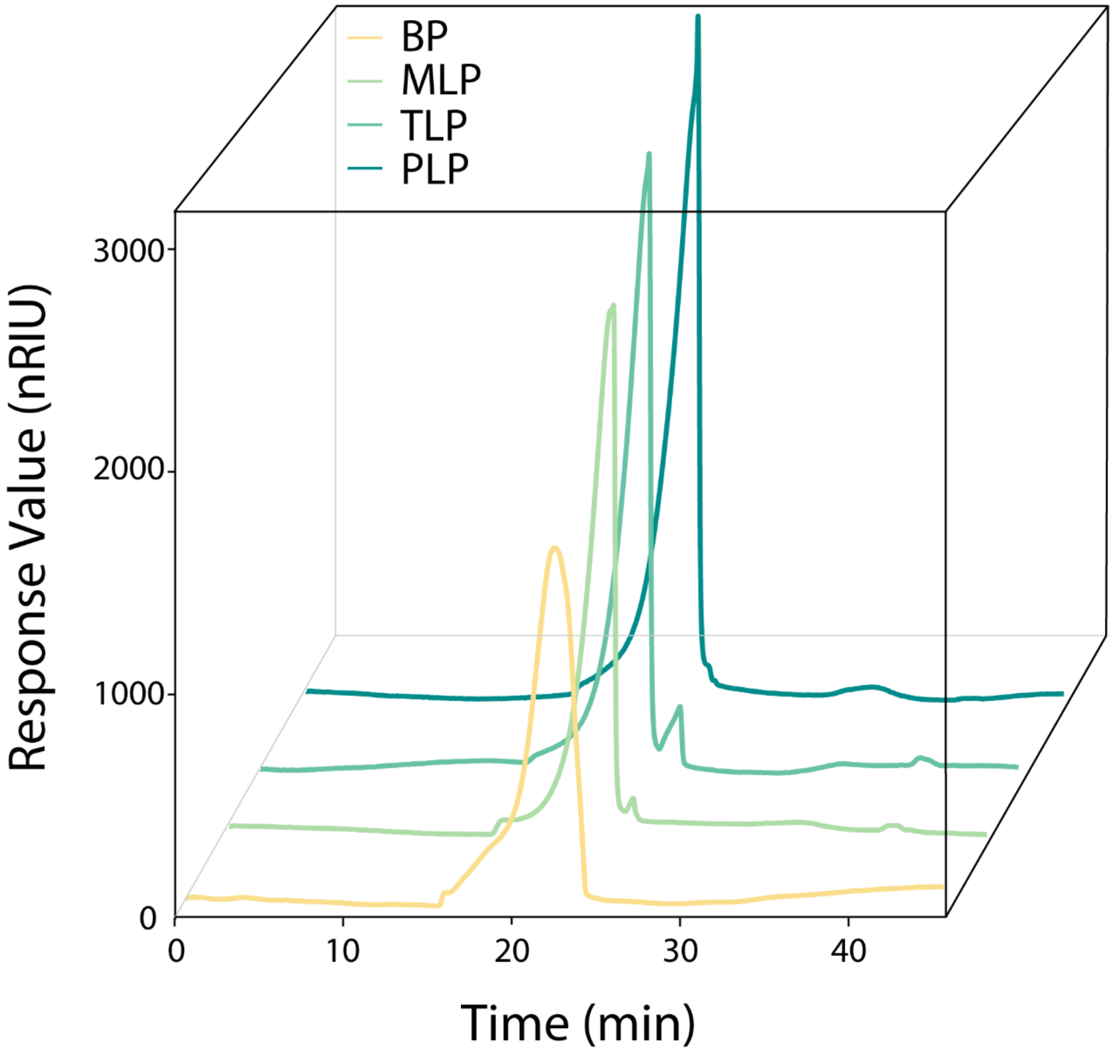

Figure S1. Peak time diagram of BP and LPs.

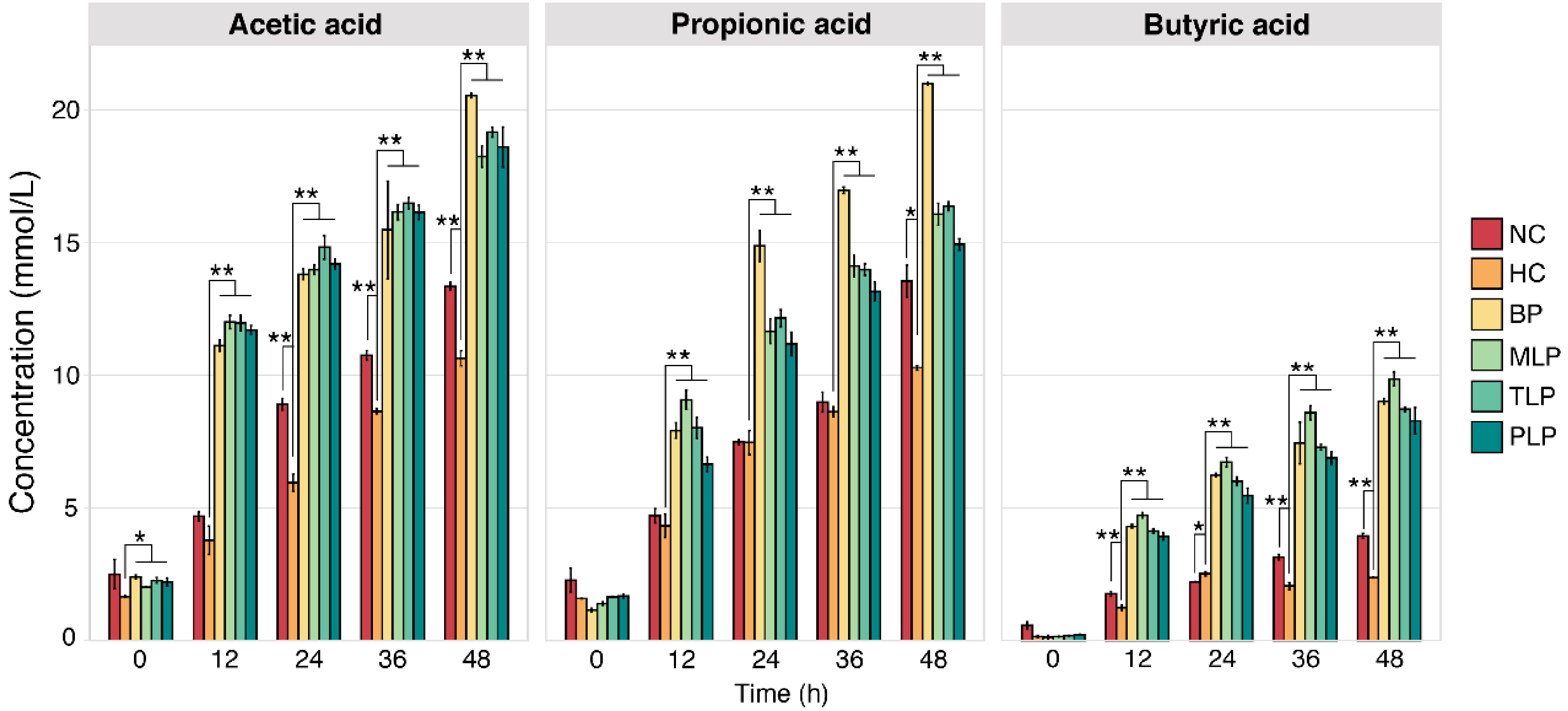

Figure S2. The levels of acetic, propionic acid and butyric acid in different medium and time during in vitro fermentation. Differences from the HC group were assessed using analysis of Student's t test and are denoted as follows: *$p < 0.05$, **$p < 0.01$.

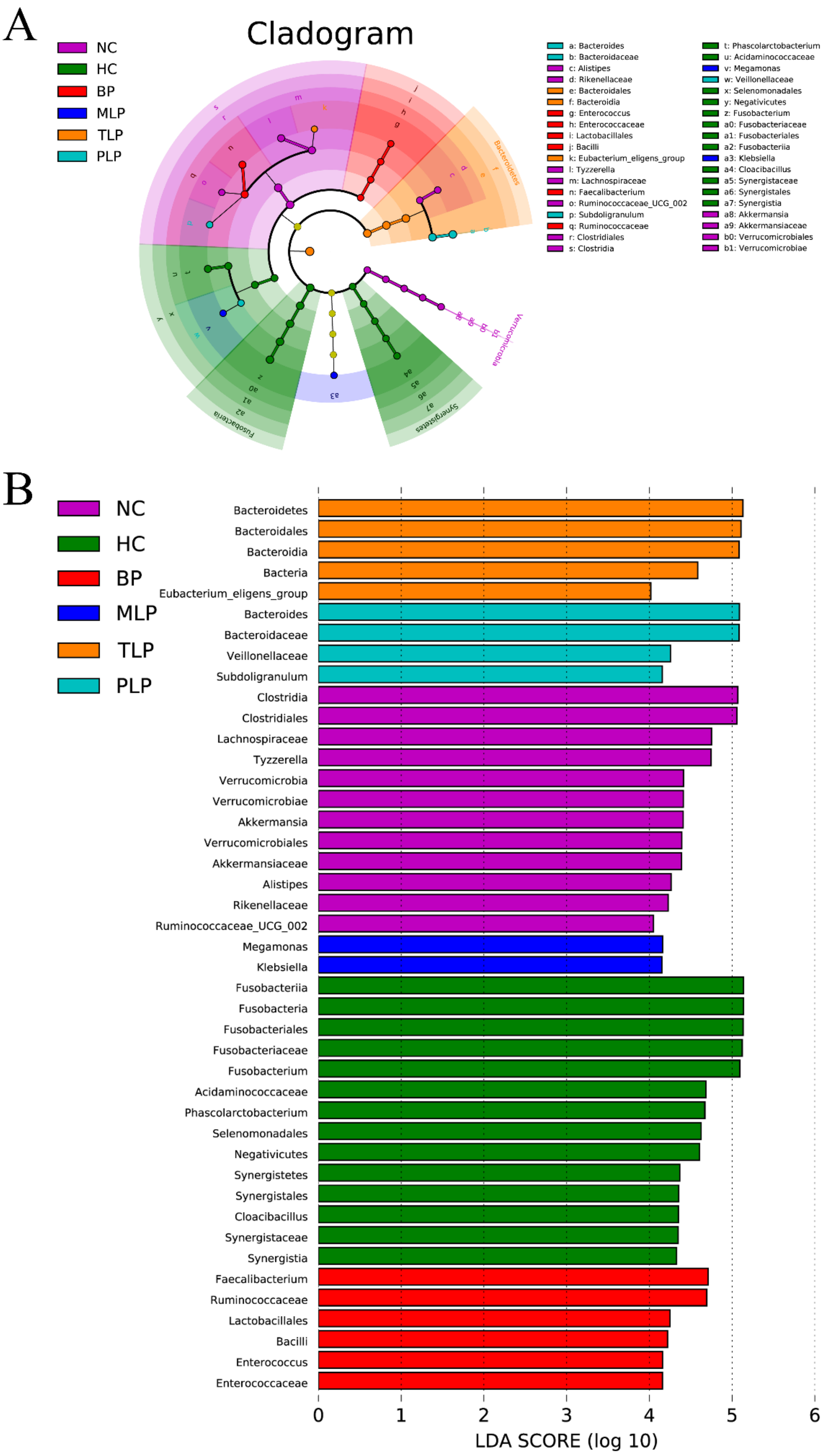

Figure S3. Characterization of microbiomes in six groups by LEfSe analysis and LDA. (A) Taxonomic representation of statistically and biologically consistent differences in six groups. (B) Histogram shows the LDA scores (log10) identified size of differentiation between different groups with a threshold value of 4.

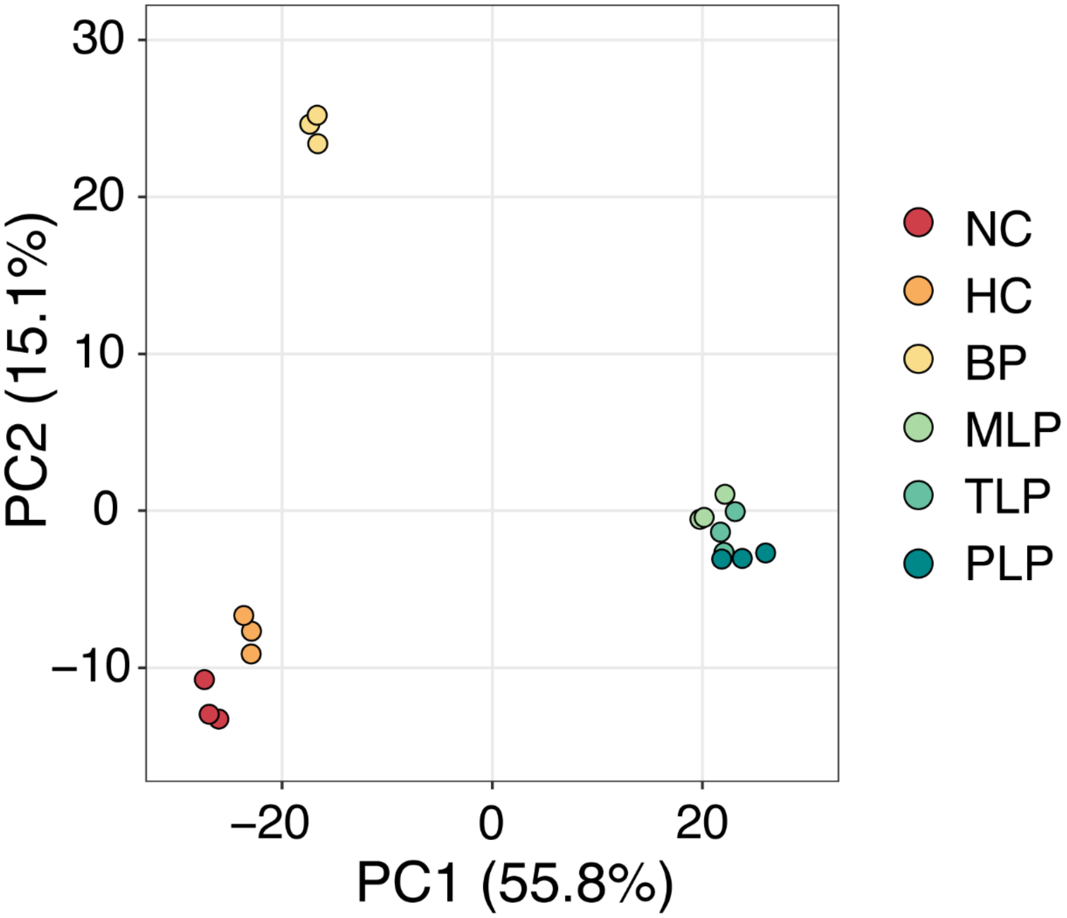

Figure S4. Analysis of PCA differences in metabolomics after fermentation to 48 hours.

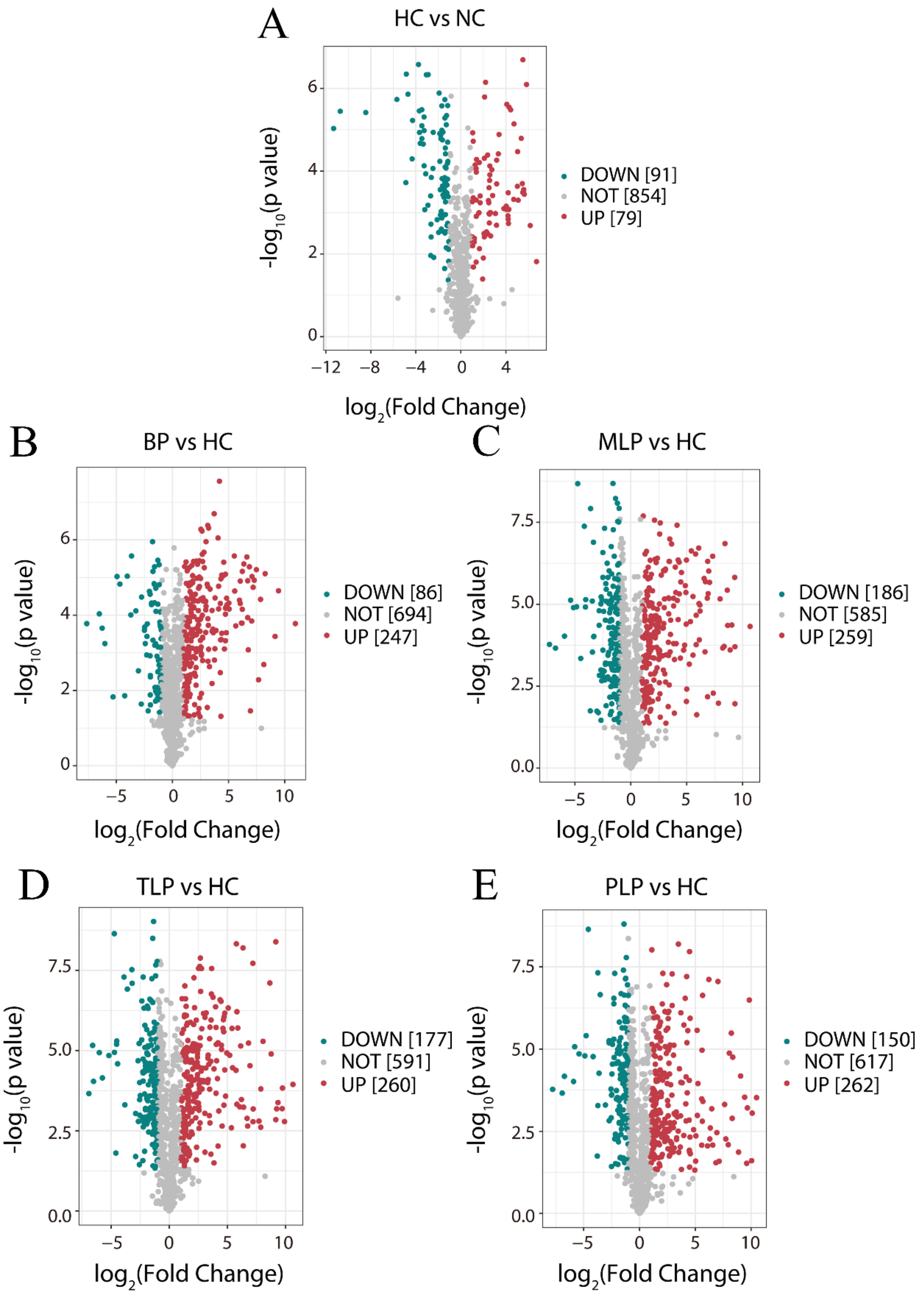

Figure S5. Volcano plot enabling the visualization of differential metabolites between different groups. (A) HC vs NC. (B) BP vs HC. (C) MLP vs HC. (D) TLP vs HC. (E) PLP vs HC. Metabolites with a fold change of <0.5 and a *p* value of <0.05 (highlighted in green) were denoted as significantly downregulated metabolites, while metabolites with a fold change of >2 and a *p* value of <0.05 (highlighted in red) represented significantly upregulated ones.
